# The Natural History of a Musculoskeletal Clinical Researcher

**DOI:** 10.5334/jbsr.2523

**Published:** 2021-09-21

**Authors:** Nawal Zia, Scott Evans, Christine Azzopardi, Steven James, Rajesh Botchu

**Affiliations:** 1 Medical School, College of Medical and Dental Sciences, University of Birmingham, Birmingham B15 2TT, UK; 2 Royal Orthopedic Hospital, Birmingham, GB

**Keywords:** natural, history, clinical researcher

## Abstract

**Background:**

Clinicians face several barriers to career progression in academia. The natural career progression of acclaimed researchers in musculoskeletal medicine will be analysed to provide a road map for a future researcher.

**Methods:**

A retrospective PubMed database search for 32 UK-based researchers in musculoskeletal medicine (orthopaedic surgeons, musculoskeletal radiologists, and rheumatologists) was performed. Researchers with over 10 years in research since their primary medical qualification were included in the study. The number of publications per year were analysed for each researcher.

**Results:**

Consultants published a median of 98 papers during their working career and a range of 51 to 399. It took a median of nine years for the first paper to be published (range = 1–27 years). It took a median number of 15 years for a clinician to reach their first peak (range = 6–34). The median number of years spent at the “peak” of one’s research career was 2 (range = 1–24).

**Conclusions:**

This is the first study to provide a road map for a musculoskeletal clinical researcher. This study identifies a need for structural change to support and encourage clinicians to participate in research. This can be introduced through a range of methods such as mentorship schemes, medical school workshops, and academic training programmes.

## Background

The demand for clinical research is increasing exponentially with the advent of new therapies, investigations, and treatment options available to the patient population. Therefore, the onus lies upon experts in the field to contribute to the development of evidence-based guidelines through original research [1]. Clinical research is a critical way to feedback to the wider health community and improve outcomes, whether it be through testing the efficacy of novel treatments, diagnosis aids, or via qualitative research. Despite this need, clinicians face several barriers to their career progression that should integrate with academia, such as lack of formal education in research, time, and experience in statistical analysis [2]. Clinicians value the rewards and skills that academic medicine can provide, such as teaching opportunities and intellectual challenges of research. A recent cross-sectional study found that over 66% of clinicians were involved in research, yet less than 20% had formal training [3]. Through literature analysis, it was observed that with one’s academic career progression that the clinical researcher is more likely to become demotivated due to questionable availability of funding, a lack of cultural and gender diversity amongst the research community, and issues regarding work-life balance [4].

The natural career progression of 33 randomly selected researchers in the field of musculoskeletal medicine will be analysed. We will then discuss the results to provide guidance etc on the typical path in the development of an academic researcher.

## Objectives

This study aimed to review the natural progression of a career in clinical researcher.

## Method

We measured “success” in clinical research by number and density of papers published by each author per year following primary medical graduation. This allowed us to gain a gross overview of how the career of a clinical researcher progresses. For a paper to be considered at a quality high enough to be a marker of success, it should have a searchable ID on PubMed. Thirty-three randomly selected clinicians in musculoskeletal medicine (orthopaedic surgeons, musculoskeletal radiologists, and rheumatologists) from 18 centres across the United Kingdom were included in the study. Inclusion criteria was that they must have had at least 10 years of medical practice post-graduation and a minimum of 50 publications in PubMed. Information on background characteristics, such as year of graduation from medical school and additional research degrees, were also collected. A retrospective PubMed database search for each researcher was performed. Different combinations of author search terms were used, such as the surname, initials, and middle names, in order to retrieve all the PubMed papers that the researcher appeared on as an author. The place at which the name appears on the author list was deemed irrelevant for this study. The number of papers written per year since their year of qualification from medical school was collated, and the number of papers published per year was also recorded. We subsequently analysed the number of papers published per year, over the lifespan of their career, from year of qualification to the present. We defined a “peak” as a year where more than 10 papers were published.

## Results

**Figure 1:**
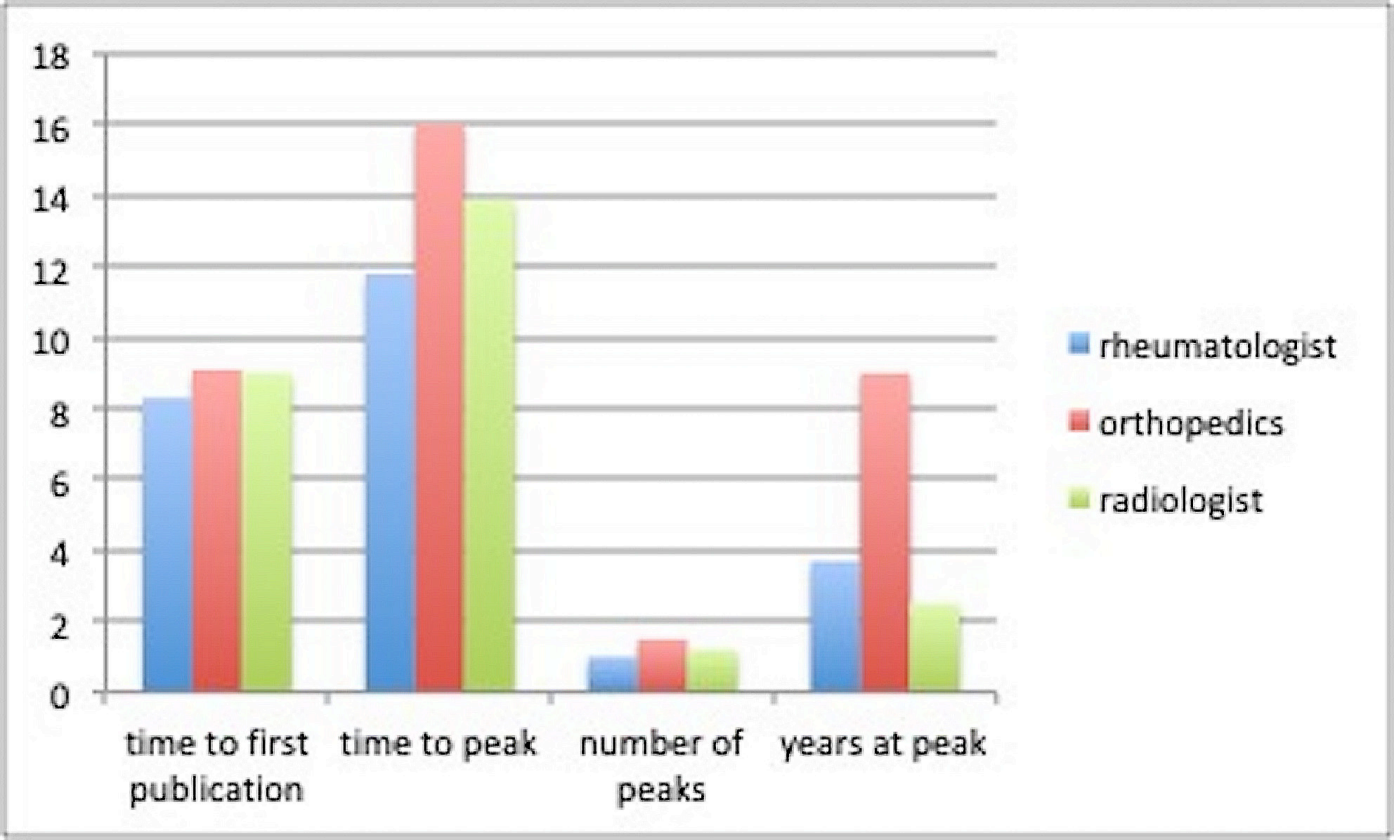
Graph showing time to publication time to peak, number of years to peak, and number of years at peak for orthopedic surgeons, radiologists, and rheumatologists in the study.

Eleven radiologists, 13 orthopaedic surgeons, and 9 rheumatologists from 18 centres were included in the study. (***Table 1, Figure 1***). 30 out of 33 clinicians had further research degrees. Twelve had PhDs, eight had master’s qualifications, and seven had bachelor’s degrees in research-based subjects. Consultants published a median of 98 papers during their working career and a range of 51 to 399. 32 consultants are still working, and one retired. Results were grouped according to specialty and analysed.

**Table 1:** Number of publication, years at peak and number of peaks for each clinician.


UNIQUE IDENTIFIER	SPECIALTY	NUMBER OF YEARS IN RESEARCH	TOTAL NUMBER OF PAPERS PUBLISHED	YEARS AFTER GRADUATION UNTIL FIRST PUBLICATION	YEARS UNTIL FIRST “PEAK”	NUMBER OF “PEAKS” IN CAREER	NUMBER OF YEARS DURING LONGEST “PEAK” OF CAREER

**Ra1**	Radiology	27	61	11	18	1	1

**Ra2**	Radiology	36	313	1	14	4	8

**Ra3**	Radiology	22	88	2	15	1	1

**Ra4**	Radiology	24	97	0	10	2	1

**Ra5**	Radiology	30	126	8	21	2	1

**Ra6**	Radiology	29	51	11	NA	0	NA

**Ra7**	Radiology	44	75	27	41	1	2

**Ra8**	Radiology	33	70	33	NA	0	NA

**Ra9**	Radiology	23	109	3	18	1	2

**Ra10**	Radiology	23	102	8	20	1	3

**Ra11**	Radiology	42	155	10	NA	0	NA

**Ortho1**	Orthopaedics	38	187	10	22	2	8

**Ortho2**	Orthopaedics	32	189	14	18	2	9

**Ortho3**	Orthopaedics	17	68	4	13	1	1

**Ortho4**	Orthopaedics	14	91	3	6	2	2

**Ortho5**	Orthopaedics	36	56	8	NA	0	NA

**Ortho6**	Orthopaedics	23	128	1	15	2	4

**Ortho7**	Orthopaedics	29	114	12	22	3	1

**Ortho8**	Orthopaedics	30	247	12	16	2	12

**Ortho9**	Orthopaedics	41	191	8	34	1	6

**Ortho10**	Orthopaedics	44	310	12	21	3	15

**Ortho11**	Orthopaedics	33	96	9	NA	0	NA

**Ortho12**	Orthopaedics	12	83	11	19	1	6

**Ortho13**	Orthopaedics	25	151	8	18	1	8

**Rheum1**	Rheumatology	32	72	16	31	1	1

**Rheum2**	Rheumatology	15	64	1	12	1	1

**Rheum3**	Rheumatology	32	57	17	NA	1	NA

**Rheum4**	Rheumatology	21	99	4	16	3	2

**Rheum5**	Rheumatology	18	103	0	NA	0	NA

**Rheum6**	Rheumatology	19	61	5	17	1	17

**Rheum7**	Rheumatology	19	270	0	10	1	10

**Rheum8**	Rheumatology	32	92	13	NA	0	NA

**Rheum9**	Rheumatology	47	399	29	30	2	15


The median time it took for a radiologist to publish their first paper was eight years (range = 1–27 years), and it took a median of 19.5 years to reach a “peak” of over 10 papers per year for those who had a peak (range = 10–41 years). The median number of papers published by radiologists were 93 and ranged from 51 to 313 during a working career. Only three out of the 11 radiologists demonstrated the typical “rise, peak, and then decline” pattern earlier discussed (27%) (Ra1, Ra3, Ra6). 2 radiologists (Ra8, Ra9) demonstrated an exponential rise. The rest of the cohort demonstrated no pattern in their career trajectories. An average radiologist in this cohort would publish 99 papers over the course of their career (median = 97, range = 51–313).

Orthopaedic surgeons took around the same median time to publish their first paper (9 years, range = 1–14 years). However, out of the cohort, clinicians in this specialty took the longest to peak, with a median of 17 years (range = 6–23 years). The number of years spent at the peak was longer, at an average of nine years. Five out of 13 orthopaedic surgeons followed the standard “peak-and-decline” career trajectory (Ortho2, Ortho3, Ortho5, Ortho9, Ortho10). Four orthopaedic surgeons demonstrated a similar pattern, but with multiple peaks ranging from 2 to 3 (Ortho1, Ortho6, Ortho7, Ortho8). 3 orthopaedic surgeons demonstrated a slow rise with no decline during their career (Ortho11, Ortho12, Ortho13). Orthopaedic surgeons published the most papers during their academic career, at a mean of 128 papers per person, ranging from around 63 to 310 in the cohort.

Rheumatologists took the shortest time to publish their first paper and to reach their first peak at a median of 5 years (range = 0–29 years) and 16.5 years respectively (range = 4–31 years). The mean number of peaks for the cohort was 1 (range = 0–3) and lasted for 1 year (range = 1–3). Rheumatologists did not demonstrate a pattern to their academic career trajectories.

## Discussion

It is important that research is published regularly in order to provide up-to-date, high-quality care to our patients. The role of the doctor has expanded to encompass the ability to not only critically appraise evidence, but to also contribute to these advancements. This is the first study to provide the roadmap the academic career pathway of a musculoskeletal researcher.

Consultants published a median of 98 papers during their working career and a range of 51 to 399. It took a median number of 15 years for a clinician to reach their first peak (range = 0–34). The median number of years spent at the “peak” of one’s research career was 2 (range = 0–24). It took a median of nine years for the first paper to be published (range = 0–27 years). For nine researchers, the typical career trajectory followed an upward trend and peak, followed by a decline. For those who start their research career earlier, their peaks tend to last longer. Whether the typical career trajectory follows a peak and subsequent decline is questionable based on our findings. This requires further in-depth analysis to determine the factors behind this.

Our study provides evidence to support the hypothesis that there is difficulty for a clinical researcher to publish their first paper. However, research career trajectories are too varied to generalise and are dependent on a range of factors, including personal preference. The study also finds some key differences in the career trajectories in different specialties, namely, orthopaedic surgeons as compared to rheumatologists, as described in the results. Perhaps certain specialties such as orthopaedics provide more opportunity for research compared to other specialties.

There are various ways to encourage more clinicians to undertake a career in academic research. This may in turn reduce the difficulty in the publication of one’s first paper, as they start their work in research much earlier. A systematic review conducted by Straus et al. found that research opportunities for medical students, mentorship schemes, and incentives were all important factors in impacting the decision-making process as to whether one would consider a career in academia [5]. A literature review assessed why and when physicians choose careers in academic medicine and found that core values as to why one must enter academic medicine are important in decision making, which can be reinforced with “research-oriented [teaching] programmes” [6]. These interventions not only can encourage clinicians in specialised tertiary centres to consider research alongside their clinical duties, but also can accelerate success via the number of published papers per year, projects, and guideline production.

Our research has found that it takes a mean time of nine years for a clinical researcher to publish their first paper. This is in line with the findings in other studies, which had found that the mean time of publication of protocol paper was 6.4 years and 7.1 years for a main paper after receiving funding [7]. The reasons for this delay are varied according to the literature. When submitting a manuscript for the first time, there is a high chance of rejection, probably due to flaws in the methods section of their paper and study design [8]. Therefore, the lag in publication of one’s first paper may be attributable to overcoming the process of reviewing and rewriting the paper in order to comply with the relevant journal’s guidelines. This may explain why 60% of the cohort had published a case report on their own or alongside original research as their first paper rather than from clinical trials. After this process, it seems to become easier for a researcher to draft publications that are focused and relevant to the target journals, leading to a rise in the number of publications per year.

Our study found that the natural progression of one’s research career is a lot more varied and dependent on several factors. We assessed some of them according to the information we had about each clinical researcher and found that the first few years after graduation from medical school, where publication numbers are the lowest, correlates with the same time that researchers were studying towards qualifications such as college memberships. Considering that upon graduation of medical school seems to be the time where “research success” tends to be lowest, it is recommended that schemes and incentives to encourage medical students to enter the clinical research pathways should be implemented as early as possible. Further research is needed in order to affirm this. Furthermore, changing priorities plays a part in how much a clinician engages in research, for example, due to exams and personal priorities, the number of papers published may drop. Conversely, retirement from medical practice may increase the number of papers published due to more disposable time.

It is important to note the baseline characteristics of our population. The cohort are all male except one and therefore the study does not take account of female researchers and their career trajectories. A recent study has found that women are significantly more likely than men to pursue an academic career, yet the results of a cross-sectional study, with a sample size of 800 women stipulated that many women feel that motherhood and pregnancy can slow the progress of their career in academic medicine [9].

The culture behind academic medicine has changed drastically over the last 20 years, with the recent introduction of initiatives such as mentorship. It is important to measure the impact of such initiatives on career development and the extent to which they encourage young clinicians to pursue academia in specialised fields. A systematic review found that mentorship can potentially play a large part in career guidance, but recommended that further evidence-based research is needed before concluding the beneficial effects in academic medicine [10]. It is recommended that further studies be conducted to assess the efficacy of workshops, mentorship schemes, and research initiatives in medical school to encourage future clinicians to pursue academic medicine before they are funded and implemented.

To summarise, our results show a varied career trajectory for each researcher, a greater insight into research demonstrated by orthopaedics, and that it takes many years for a clinician to publish their first paper post-graduation.

## Limitations

Only 33 clinical researchers were analysed, and this is not representative of all clinical researchers. We had only conducted a PubMed search to retrieve the published papers. This could introduce publication bias as there may have been additional papers published without a PubMed ID or predated before the earliest date we have searched. The entire cohort of researchers were male except one (Rheum 8). This is not representative of the career progression of both genders. Furthermore, PubMed ID’d papers may not be an accurate representation of career success in academic research. For example, we did not take account of other markers of success such as conference presentations, publications in non-PubMed ID’d journals, and honorary lectures. Since we did not take account of the place in the author list that the researcher appeared, some papers may have the researcher’s name on, but the amount of contribution is not considered. There are other factors that could indicate research success that were not taken account of in the study, such as the impact factor of the journal by which each paper was published or the number of times an article was cited.

Therefore, we can only take our results as a crude indicator of research success, and we recommend detailed analysis in further studies.

## Conclusion and recommendations

Assessing the career trajectories of specialised consultants indicates a slow academic career progression, even for those who are now highly acclaimed. This can be attributed to a range of factors mentioned previously, such as lack of education or insight into the benefits of research and manuscript rejections.

This preliminary study identifies a need for structural change to encourage and support clinicians into research and provide guidance in the publication of their first paper. This can be introduced through a range of methods, such as mentorship schemes, medical school workshops, and programmes such as the academic foundation training post.

It is recommended that a survey be distributed to clinicians in different hospitals, to ascertain the reasons why they would/would not want to partake in research alongside their career and what barriers might be holding them back. The results can then be analysed, and new initiatives can be piloted to make research pathways more accessible to clinicians.

Even from the results of this study, there is a call for hospitals to implement teaching programmes, organise conferences, and conduct courses in publishing research, especially in specialist centres, whereby clinical researchers are supported from graduation to the post-retirement period. This will increase the number of researchers in specialised centres and therefore will multiply the output of regularly updated guidelines supported by evidence-based medicine.

### Main message

- Consultants published a median of 98 papers during their working career and a range of 51 to 399.
- It took a median of nine years for the first paper to be published (range = 1–27 years).
- This is the first study to provide a road map of a musculoskeletal clinical researcher.
